# Marked variations in gut microbial diversity, functions, and disease risk between wild and captive alpine musk deer

**DOI:** 10.1007/s00253-023-12675-1

**Published:** 2023-07-08

**Authors:** Feng Jiang, Pengfei Song, Daoxin Liu, Jingjie Zhang, Wen Qin, Haijing Wang, Chengbo Liang, Hongmei Gao, Tongzuo Zhang

**Affiliations:** 1 Present Address: Key Laboratory of Adaptation and Evolution of Plateau Biota, Northwest Institute of Plateau Biology, Chinese Academy of Sciences, 23 Xinning Rd, Chengxi District, Qinghai 810001 Xining, China; 2 Qinghai Provincial Key Laboratory of Animal Ecological Genomics, Xining, 810001 Qinghai China; 3 University of Chinese Academy of Sciences, Beijing, 100049 China; 4 State Key Laboratory of Plateau Ecology and Agriculture, Qinghai University, Xining, 810016 Qinghai China; 5 College of Agriculture and Animal Husbandry, Qinghai University, Xining, 810016 Qinghai China

**Keywords:** Alpine musk deer, Core microbiome, Opportunistic pathogens, 16S rRNA gene sequencing, Gut microbial function

## Abstract

**Abstract:**

Maintaining a healthy status is crucial for the successful captive breeding of endangered alpine musk deer (*Moschus chrysogaster*, AMD), and captive breeding programs are beneficial to the ex-situ conservation and wild population recovery of this species. Meanwhile, the gut microbiota is essential for host health, survival, and environmental adaptation. However, changes in feeding environment and food can affect the composition and function of gut microbiota in musk deer, ultimately impacting their health and adaptation. Therefore, regulating the health status of wild and captive AMD through a non-invasive method that targets gut microbiota is a promising approach. Here, 16S rRNA gene sequencing was employed to reveal the composition and functional variations between wild (*N* = 23) and captive (*N* = 25) AMD populations. The results indicated that the gut microbiota of wild AMD exhibited significantly higher alpha diversity (*P* < 0.001) and greater abundance of the phylum *Firmicutes*, as well as several dominant genera, including *UCG-005*, *Christensenellaceae R7 group*, *Monoglobus*, *Ruminococcus*, and *Roseburia* (*P* < 0.05), compared to captive AMD. These findings suggest that the wild AMD may possess more effective nutrient absorption and utilization, a more stable intestinal microecology, and better adaption to the complex natural environment. The captive individuals displayed higher metabolic functions with an increased abundance of the phylum *Bacteroidetes* and certain dominant genera, including *Bacteroides*, *Rikenellaceae RC9 gut group*, *NK4A214 group*, and *Alistipes* (*P* < 0.05), which contributed to the metabolic activities of various nutrients. Furthermore, captive AMD showed a higher level of 11 potential opportunistic pathogens and a greater enrichment of disease-related functions compared to wild AMD, indicating that wild musk deer have a lower risk of intestinal diseases and more stable intestinal structure in comparison to captive populations. These findings can serve as a valuable theoretical foundation for promoting the healthy breeding of musk deer and as a guide for evaluating the health of wild-released and reintroduced musk deer in the future.

**Key points:**

*• Wild and captive AMD exhibit contrasting gut microbial diversity and certain functions.*

*• With higher diversity, certain bacteria aid wild AMD’s adaptation to complex habitats.*

*• Higher potential pathogens and functions increase disease risk in captive AMD.*

## Introduction

China holds the distinction of having the world’s largest and most widely distributed population of musk deer, comprising over 70% of the global total while also contributing to more than 90% of the world’s total musk yield (Sun et al. 2018). Musk, secreted by adult male deer, is a highly valuable traditional Chinese medicine and natural flavoring agent, prized for its high medical and economic worth (Tang et al. 2018). Long-term illegal hunting and habitat fragmentation have caused the wild musk deer population in China to decline rapidly by more than 97% over the past 70 years, from an estimated 2–3 million in the 1950s to approximately 73,480 in 2009 (Wu and Wang 2006; National Forestry and Grassland Administration 2009). Of the six species of musk deer in China, the alpine musk deer (*Moschus chrysogaster*, AMD) is the largest. The population of wild AMD in China is estimated to be around 28,000 and is classified as endangered (EN) on the IUCN Red List and critically endangered (CR) on the Red List of Vertebrates of China (Jiang et al. 2022), mainly distributed in the Qinghai-Tibet Plateau and surrounding areas in China, including high-altitude meadows, shrublands or coniferous forests in southeastern Tibet, Qinghai, Qilian Mountains and in Xinglong mountain of Gansu, Helan Mountains in Ningxia, and western Sichuan and northern Yunnan (Jiang et al. 2020; Bao et al. 2020). Currently, successful captive breeding in AMD serves as the foundation for both the wild release and sustainable utilization of musk deer resources.

Captive breeding programs for musk deer in China were initiated in 1958 as an alternative to killing musk deer for obtaining their highly valued musk (Yang et al. 2003), which has been considered an effective measure for ex-situ conservation of wild musk deer resources and sustainable utilization of musk. Over the last six decades, the scale of captive breeding of musk deer, particularly of the AMD, has gradually expanded, establishing it as the second-largest artificial musk deer breeding program in China. The Gansu Xinglong Mountain National Nature Reserve, which began domesticating musk deer in the 1990s, currently holds the largest musk deer farm in China (Meng et al. 2008; Sun 2018). Despite its potential advantages, captive breeding of musk deer is not without challenges. Compared to their wild musk deer, captive individuals exhibit a heightened susceptibility to intestinal diseases, including gastroenteritis, diarrhea, and gastrointestinal bleeding, which are often linked to bacterial dysregulation, consequently resulting in elevated mortality rates and constituting the primary limiting factor for the expansion of artificial breeding scales of musk deer (Qiao et al. 2009).

Gut microbiota is a critical component of the host intestinal microecology, performing a vital function in maintaining intestinal homeostasis, promoting host health, and modulating physiological processes such as metabolism, nutrient absorption, growth, and development, as well as immune regulation and resistance to foreign pathogens (Alberdi et al. 2016; Nicholson et al. 2012; Schluter et al. 2020). Differences in gut microbiota between wild and captive populations of mammals can significantly impact the overall digestive and immune functions of the host (Gibson et al. 2019). Studies investigating gut microbiota of captive and free-ranging Namibian cheetahs (*Acinonyx jubatus*) demonstrated that alpha diversity of gut microbiota did not differ significantly, but captive Namibian cheetahs showed a higher abundance of operational taxonomic units (OTUs) and disease-related functional pathways linked to potential pathogens (Wasimuddin et al. 2017). Wild snub-nosed monkeys (*Rhinopithecus brelichi*) exhibited higher diversity of gut microbiota and plant-degrading microbiota, while captive individuals had higher carbohydrate-degrading and mucin-degrading microbiota, suggesting that changes in food and microbiota may lead to decreased health of captive individuals (Hale et al. 2019). Therefore, we contend that leveraging gut microbiota diversity and function can improve the health status and management strategies for captive musk deer. Analyzing the differences in gut microbial composition and function between captive and wild musk deer is a crucial measure for promoting the wild release and population expansion of the species.

In this study, the 16S rRNA gene sequencing was utilized to approach to investigate the composition and diversity of gut microbiota in captive and wild musk deer at various taxonomic levels. The key scientific questions addressed in this study were (*i*) identifying the core microbiota of musk deer; (*ii*) determining the variation in gut microbial composition between captive and wild musk deer; (*iii*) analyzing the differences in dominant bacteria and metabolic function; and (*iv*) investigating changes in opportunistic pathogens and disease-related function between wild and captive musk deer. The results of this study will provide a scientific basis for developing effective health management strategies for musk deer by analyzing differences in gut microbiota between captive and wild populations.

## Materials and methods

### Sampled materials

**Fig. 1:**
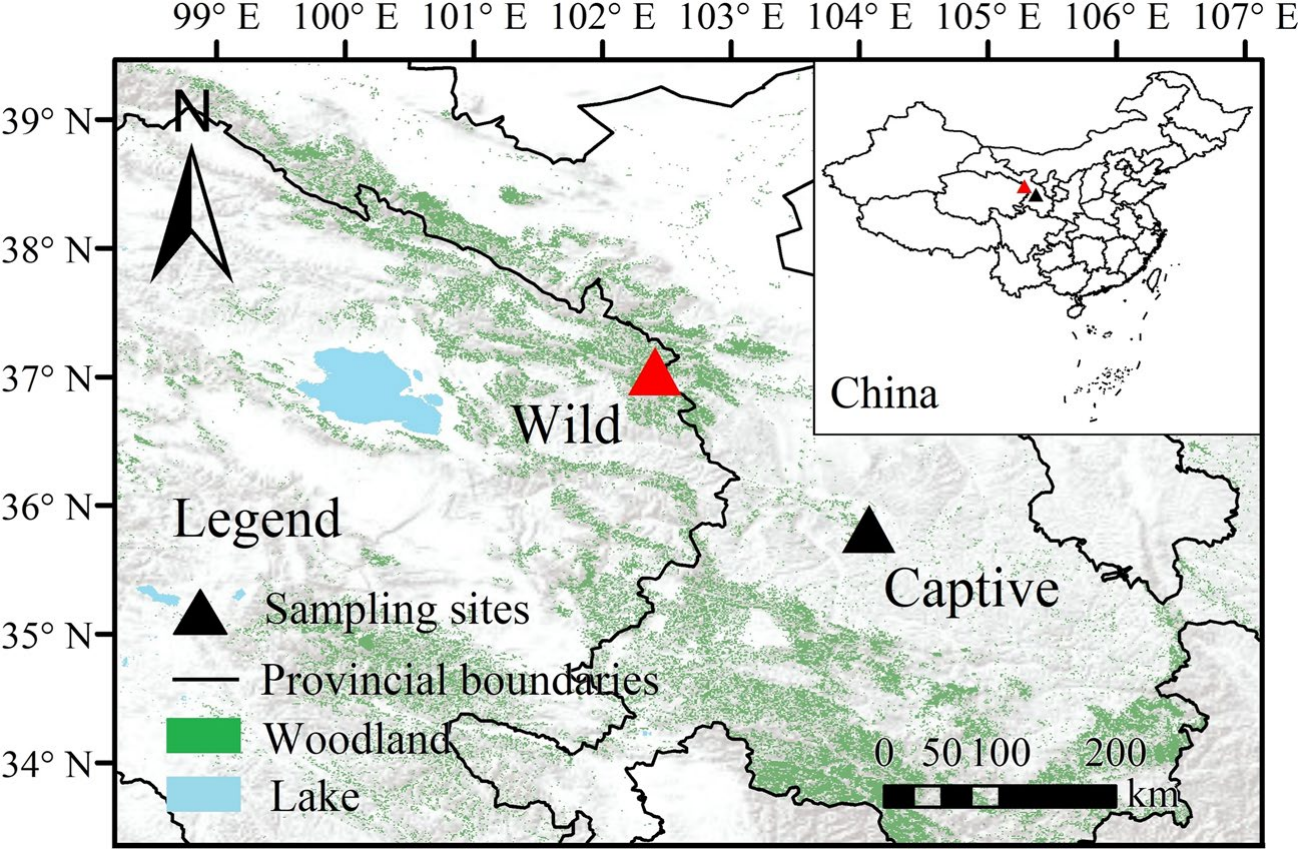
Sampling sites of wild (red triangle) and captive (black triangle) alpine musk deer (AMD)

A total of 23 fresh fecal samples were collected from wild AMD using non-invasive sampling methods on March 5 and 6, 2021, at Beishan National Forest Park in Huzhu County, Qinghai Province (Fig. 1). Additionally, 25 fecal samples were collected from captive individuals on March 10 and 11, 2021, at the AMD breeding center, located in Xinglong Mountain National Nature Reserve in Yuzhong County, Gansu Province.

Given the relatively consistent locations of wild AMD resting sites and manure fields, it is advantageous to procure relatively fresh fecal samples from wild individuals in close proximity to these areas. Prior to sampling the captive individuals, the AMD breeding houses underwent a thorough cleaning, and each individual was confined to an individual breeding house to enable the collection of fresh feces the following morning. During the sampling process, fresh fecal samples were collected immediately after defecation using sterile disposable polyethylene gloves and sterile sampling bags.

Following labeling, all samples were stored in a − 20℃ vehicle-mounted refrigerator. Upon arrival at the laboratory, the samples were either processed immediately or stored in a − 80 ℃ ultra-low temperature refrigerator for subsequent DNA extraction.

### DNA extraction and 16S rRNA gene sequencing

Fresh fecal samples from musk deer weighing 200 mg were aseptically collected and transferred to 2-mL sterile centrifuge tubes. The E.Z.N.A.® Soil DNA Kit from Omega Bio-Tek (Norcross, GA, USA) was used to extract genomic DNA from both wild and captive AMD fecal samples in accordance with the manufacturer’s instructions. The quality of DNA extraction was assessed using 1% agarose gel electrophoresis, while the purity of DNA was determined using the NanoDrop2000 (Thermo Fisher Scientific, Waltham, MA, USA).

The V4 and V5 hypervariable regions of the 16S rRNA gene were amplified via PCR using the following primers: forward primer 515F (5’-GTGCCAGCMGCCGCGG-3’) and reverse primer 907R (5’- CCGTCAATTCMTTTRAGTTT-3’). Each PCR reaction contained 4 μL of TransStart FastPfu buffer (5 ×), 2 μL of dNTPs (2.5 mM), 0.8 μL of each primer (5 μM), 0.4 μL of TransStart FastPfu DNA polymerase, 10 ng of template DNA, and ddH_2_O to make up to 20 μL. The PCR reaction was performed under the following conditions: initial denaturation for 3 min at 95 ℃, followed by 27 cycles of denaturation for 30 s at 95 ℃, annealing for 30 s at 55 ℃, extension for 45 s at 72 ℃, and final extension for 10 min at 72 °C.

### Operational taxonomic unit (OTU) clustering and taxonomic annotation

The PCR products were extracted using 2% agarose gel electrophoresis and purified with the AxyPrep DNA Gel Extraction Kit (Axygen Biosciences, Union City, CA, USA). The purified PCR products were analyzed by 2% agarose gel electrophoresis and quantified using a Quantus™ Fluorometer (Promega, Madison, WI, USA). The NEXTflex® Rapid DNA-Seq Kit (Bioo Scientific, Austin, TX, USA) was used to prepare libraries for sequencing. The joint linking of the PCR amplified fragments was performed in series, and magnetic bead screening was used to remove self-connecting sequence fragments. The resulting libraries were purified and recovered for sequencing on an Illumina Miseq PE300 platform (Illumina, San Diego, CA, USA). The raw sequencing data were deposited in the NCBI SRA database (https://dataview.ncbi.nlm.nih.gov/object/PRJNA753621?reviewer=cqgndbrcp4dkppi39a3mid6tef).

To process the raw sequencing data, Trimmomatic software (v. 0.39) was used to filter out low-quality bases with an end mass score below 20 (Bolger et al. 2014), and the paired-end reads were merged using Flash software (v 1.2.7) (Magoč and Salzberg 2011). OTU clustering was performed on all samples with 97% similarity using UPARSE software (v 7.1) (Costello et al. 2009), and chimeric sequences were removed during clustering. The species classification of each valid sequence was annotated using the Ribosomal Database Project (RDP) classifier (http://rdp.cme.msu.edu/) with the Silva database (Silva 138/16S) at a threshold of 0.8 (Wang et al. 2007; Jiang et al. 2022).

### Bioinformatic analysis

After the taxonomic annotation of OTUs, the corresponding abundance information of each OTU annotation in each sample was counted, and the sample sequences were subsampled according to the minimum number of sample sequences. The abundance of all samples or each group of samples in wild and captive AMD at the phylum and genus levels was displayed using community histograms and Venn diagrams with the R software (v 3.3.1, https://cran.rstudio.com/; packages “stats”). The similarity and difference of microbial composition were analyzed, and the abundance histogram and heatmap were drawn with the R software (package “pheatmap”).

At the alpha diversity level, four indices were measured with Qiime software (http://qiime.org/scripts/assign_taxonomy): *S*_*obs*_ (the number of observed OTUs), Shannon, Chao1, and phylogenetic diversity (PD) (Caporaso et al. 2010). Based on the results of the normality test, it was determined that the α index data mentioned above did not follow a normal distribution. Consequently, the Wilcoxon rank-sum test, a non-parametric statistical method, was employed to analyze the significant differences of each Alpha diversity index among different groups with the R software (packages “stats”). Principal coordinates analysis (PCoA) was used to analyze beta diversity among different groups, with the Bray–Curtis distance used to calculate the distance between samples at the OTU level using the R software (package “vegan”). Analysis of similarities (ANOSIM), a non-parametric statistical test, was used to test the difference between groups with a two-tailed test with the R software (packages “vegan,” anosim function) (Oksanen et al. 2019). A false discovery rate (FDR) was selected to conduct multiple test corrections for the *P*-value, with a confidence level set at 0.95.

Based on the identified bacterial genera mentioned above, the information regarding potential pathogenic bacteria was primarily obtained through a literature review. Similarly, the normality test analysis revealed that the majority of the data mentioned above did not conform to a normal distribution. Therefore, the Wilcoxon rank-sum test was used to analyze the difference in the relative abundance of dominant bacteria and opportunistic pathogens between wild and captive AMD with the R software (packages “stats”). PICRUSt (phylogenetic investigation of communities by reconstruction of unobserved states) was used to standardize OTU abundance tables (Langille et al. 2013). Gene function annotation and classification analysis were performed based on KEGG (Kyoto encyclopedia of genes and genomes, http://www.genome.jp/kegg/) and eggNOG (evolutionary genealogy of genes: Non-supervised Orthologous Groups, http://eggnog.embl.de/) databases. Furthermore, the Pearson correlation coefficient of the top 50 bacterial genera in wild and captive AMD was calculated using R software (package “igraph”). Then, the co-occurrence network of the bacteria was constructed and visualized using Gephi software (v 0.9.2) (Zhang et al. 2022).

## Results

### Gut microbial community composition

After quality control, a total of 528,7059 raw reads were obtained from 48 fecal samples of musk deer, resulting in an average of 110,147 reads per sample, with an average read length of 375 bp. A total of 2105 effective OTUs were identified after clustering the reads at 97% similarity, with 1716 OTUs shared by both wild and captive AMD and 152 OTUs and 237 OTUs unique to wild and captive individuals, respectively. The identified OTUs were classified into 12 phyla, 21 classes, 52 orders, 97 families, and 212 genera. The Good’s coverage index indicated that the gut microbiota in both wild and captive musk deer was highly represented, with a coverage index of more than 99%.

**Fig. 2:**
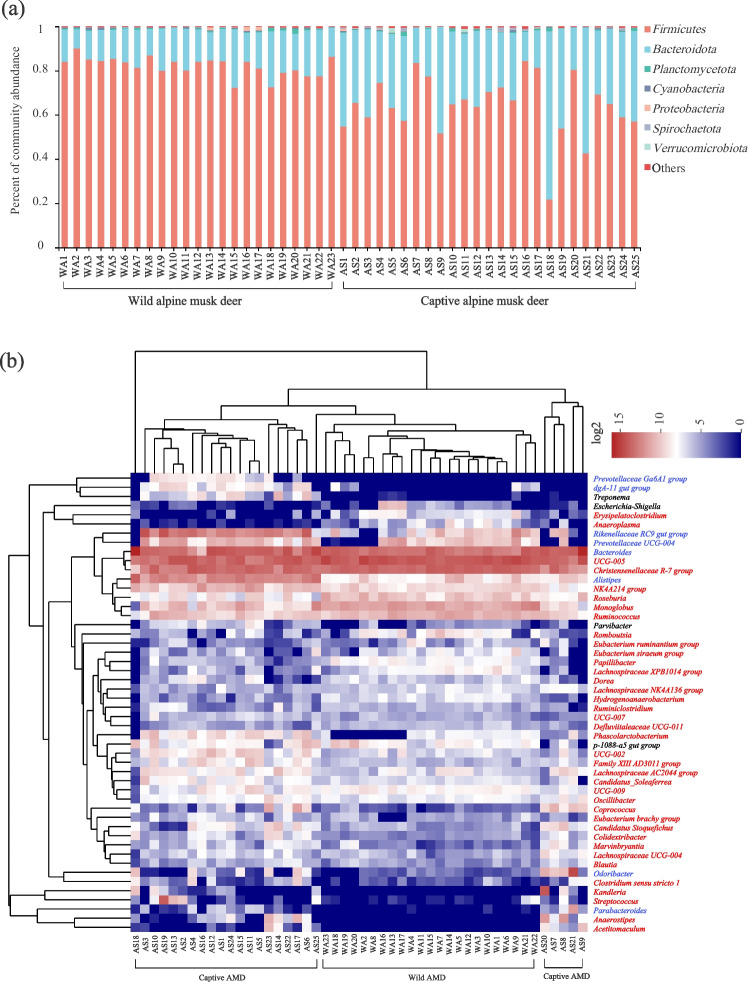
Gut microbial community composition in wild and captive AMD. (**a**) Histogram analysis of the relative abundance of phyla bacteria. (**b**) Heatmap analysis based on the top 50 genera bacteria. The color scale ranges from blue (low abundance) to red (high abundance). The red, blue, and black characters represent *Firmicutes*, *Bacteroidetes*, and other non-dominant phyla bacteria, respectively

Based on species annotation, it was found that *Firmicutes* (wild, 64.2%; captive, 82.1%) and *Bacteroidetes* (wild, 33.9%; captive, 16.3%) were the dominant phyla with a relative abundance greater than 1% in both wild and captive AMD (Fig. 2a). Among the top 50 identifiable bacterial genera, *UCG-005*, *Christensenellaceae R-7 group*, *Bacteroides*, *Monoglobus*, *Ruminococcus*, *Prevotellaceae UCG-004*, and *Rikenellaceae RC9 gut group* were the identifiable dominant genera with a relative abundance greater than 1% shared by wild and captive AMD (Fig. 2b). Additionally, other dominant genera identified in wild individuals included the genus *Roseburia*, while those in captive individuals included genera *NK4A214 group* and *Alistipes*, all belonging to the phyla *Firmicutes* or *Bacteroidetes*. The clustering results indicated that wild individuals and captive individuals clustered into one group, respectively.

### Difference analysis of gut microbiota between wild and captive AMD

**Fig. 3:**
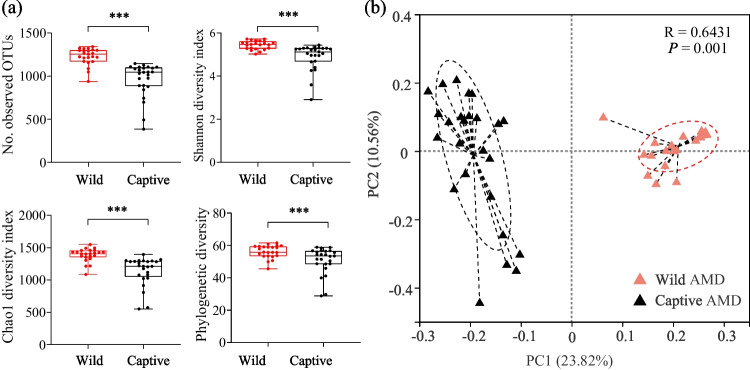
Comparison of gut microbiota diversity and composition between wild and captive AMD. (**a**) Alpha diversity analysis based on *S*_*obs*_, Shannon, Chao1, and phylogenetic diversity (PD) indices. (**b**) Principal coordinates analysis (PCoA) based on the Bray–Curtis distance matrix showing the separation between wild and captive AMD. *P < 0.05 (Wilcoxon rank-sum test), **P < 0.01 and ***P < 0.001

The α diversity of gut microbiota in wild AMD was found to be significantly higher than that in captive individuals (Fig. 3a), as measured by four alpha diversity indices: *S*_*obs*_, Shannon, Chao1, and phylogenetic diversity (PD) (Fig. 3a). The PCoA and ANOSIM also indicated a significant difference in gut microbial composition between wild and captive AMD, with an R value greater than 0 and *P* value of 0.001, respectively (Fig. 3b). These results showed that the inter-group difference in gut microbiota composition between wild and captive AMD was significantly greater than the intra-group difference.

### Differences in dominant bacteria and metabolic functions of gut microbiota between wild and captive AMD

**Fig. 4:**
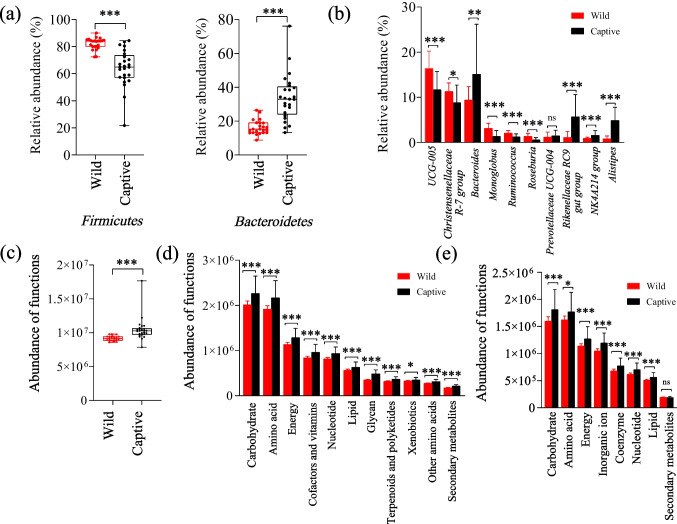
Difference analysis of dominant bacteria and metabolic function in gut microbiota between wild and captive AMD. Difference analysis of dominant phyla bacteria (**a**) and dominant genera bacteria (**b**). Difference analysis of metabolic function based on the KEGG (Kyoto encyclopedia of genes and genomes) database at level 1 (**c**) and level 2 (**d**), and EggNOG (evolutionary genealogy of genes: Non-supervised Orthologous Groups) database (**e**). *P < 0.05 (Wilcoxon rank-sum test), **P < 0.01 and ***P < 0.001. ns, no significant

There were significant differences observed in the relative abundance of both the phyla *Firmicutes* and *Bacteroidetes* between wild and captive AMD. The phylum *Firmicutes* was significantly more abundant in wild individuals than in captive individuals, while the phylum *Bacteroidetes* showed the opposite pattern (Fig. 4a). In addition, the abundance of the genera *UCG-005*, *Christensenellaceae R7 group*, *Monoglobus*, *Ruminococcus*, and *Roseburia* was significantly higher in wild AMD compared to captive individuals (*P* < 0.05) (Fig. 4b). Conversely, the abundance of the genera *Bacteroides*, *Rikenellaceae RC9 gut group*, *NK4A214 group*, and *Alistipes* was significantly higher in captive individuals compared to wild individuals (*P* < 0.05). The genus *Prevotellaceae UCG-004* did not show any significant difference in abundance between wild and captive individuals.

Based on functional annotation analysis in the KEGG database, the gut microbiota of both wild and captive AMD was found to mainly perform metabolic functions, with a focus on carbohydrate metabolism (*X̅* = 10.31%), amino acid metabolism (*X̅* = 9.85%), energy metabolism (*X̅* = 5.84%), metabolism of cofactors and vitamins (*X̅* = 4.34%), nucleotide metabolism (*X̅* = 4.22%), lipid metabolism (*X̅* = 2.87%), glycan biosynthesis and metabolism (*X̅* = 2.01%), metabolism of terpenoids and polyketides (*X̅* = 1.65%), xenobiotic biodegradation and metabolism (*X̅* = 1.62%), metabolism of other amino acids (*X̅* = 1.41%), and biosynthesis of other secondary metabolites (*X̅* = 0.93%). Functional difference analysis at level 1 and level 2 of the KEGG database indicated that the metabolic functions related to various nutriment and energy in wild AMD were significantly higher than those in captive individuals (Fig. 4c, d).

Based on functional annotation analysis using the EggNOG database, the COG functions related to metabolism were primarily associated with carbohydrate transport and metabolism $(\overline{X }=$ 8.13%), amino acid transport and metabolism $(\overline{X }=$ 8.09%), energy production and conversion $(\overline{X }=$ 5.75%), inorganic ion transport and metabolism $(\overline{X }=$ 5.35%), coenzyme transport and metabolism $(\overline{X }=$ 3.47%), nucleotide transport and metabolism $(\overline{X }=$ 3.15%), lipid transport and metabolism $(\overline{X }=$ 2.55%), and secondary metabolites biosynthesis, transport, and catabolism ($\overline{X }=$ 0.91%). While there were no significant differences in the COG function related to secondary metabolites biosynthesis, transport, and catabolism (*P* > 0.05), all other metabolic functions of various nutrients and energy in wild AMD were significantly higher than those in captive individuals (Fig. 4e).

### Opportunistic pathogens and disease-related function differences between wild and captive AMD

**Fig. 5:**
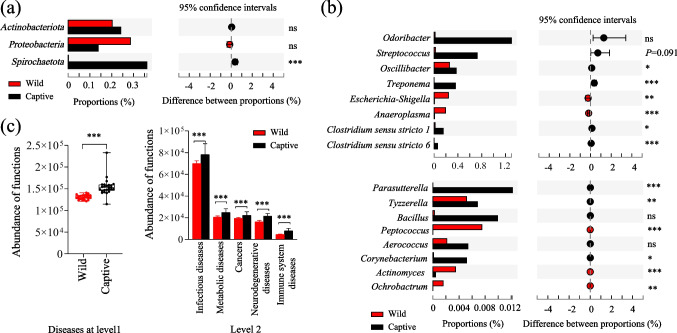
Difference analysis of opportunistic pathogens and disease-related function in gut microbiota between wild and captive AMD. (**a**) Difference analysis of opportunistic pathogens at the phylum level (**a**) and the genus level (**b**). (**c**) Difference analysis of disease-related function based on the KEGG (Kyoto encyclopedia of genes and genomes) database. *P < 0.05 (Wilcoxon rank-sum test), **P < 0.01 and ***P < 0.001. ns, no significant

The Wilcoxon rank-sum test was used to analyze the significant differences in potential opportunistic pathogens between wild and captive AMD. At the phylum level, captive individuals showed a higher relative abundance of *Actinobacteriota* and *Spirochaete* compared to wild individuals (Fig. 5a), while the relative abundance of *Proteobacteria* was higher in the wild group. At the genus level, captive individuals had a higher relative abundance of *Odoribacter*, *Streptococcus*, *Oscillibacter*, *Treponema*, *Clostridium *sensu stricto* 1*, *Clostridium *sensu stricto* 6*, *Parasutterella*, *Tyzzerella*, *Bacillus*, *Aerococcus*, and *Corynebacterium* compared to wild individuals (Fig. 5b). In contrast, the genera *Erysipelatoclostridium*, *Escherichia-Shigella*, *Anaeroplasma*, *Peptococcus*, *Actinomyces*, and *Ochrobactrum* were more abundant in the wild group.

Functional annotation analysis in the KEGG database revealed that at level 1, the enrichment of disease-related functions of gut microbiota in captive AMD was significantly higher than that in wild individuals (Fig. 5c). At level 2, the disease-related functions related to infectious diseases, metabolic diseases, cancers, neurodegenerative diseases, and immune system diseases were significantly more pronounced in captive individuals than in wild individuals (Fig. 5c).

### Co-occurrence network of the core bacteria in wild and captive AMD

**Fig. 6:**
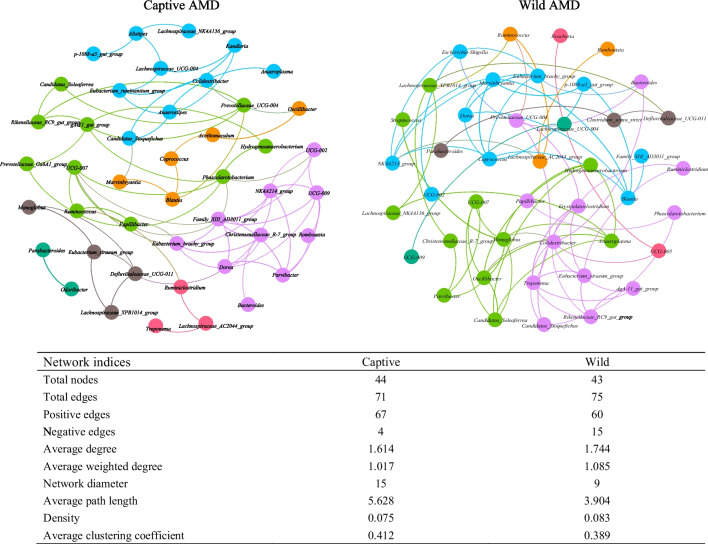
Co-occurrence networks of the top 50 bacteria genus in wild and captive AMD. Each line represents Pearson correlation coefficient (|r|≥ 0.5, *P* < 0.05)

Co-occurrence network analysis revealed differences in the complexity and stability of gut microbiota between wild and captive AMD. The wild group had more links, and the ratio of negative correlation to positive correlation was higher than that of the captive group, indicating that the gut microbiota in wild AMD was more complex and stable that in the captive AMD (Fig. 6).

Among the bacteria genera in the wild AMD, *Colidextribacter*, *Monoglobus*, *Oscillibacter*, *Treponema*, *Coprococcus*, *Eubacterium siraeum group*, and *Erysipelatoclostridium* had more correlations with other bacteria genera, suggesting their importance in the gut microbiota of wild AMD. In contrast, in captive AMD, *Family XIII AD3011 group*, *Dorea*, *NK4A214 group*, *UCG-009*, *Phascolarctobacterium*, *Papillibacter*, and *Eubacterium brachy group* had the largest number of edges, indicating their higher connectivity and potential influence in the gut microbiota of captive AMD.

## Discussions

The gut microbiota has co-evolved with the host and is an essential component of the host’s intestinal microecosystem (Lee and Mazmanian 2010), playing a crucial role in regulating host metabolism, growth, development, immune function, pathogen defense, ecological adaptation, and evolution (Nicholson et al. 2012; Schluter et al. 2020). The composition and diversity of the gut microbiota are influenced by various internal and external factors, such as age, genetic background, gender, food, season, geography, and rearing environment (Turnbaugh et al. 2009; Lozupone et al. 2012; Rao et al. 2021). Analyzing the differences in gut microbial composition and function between wild and captive AMD is essential for their successful release and population expansion in the wild. The V4-V5 variable region is considered an optimal region for analyzing the bacterial 16S rRNA gene, as it exhibits minimal genomic heterogeneity and effectively captures inter-genomic variation (Sun et al. 2013; Fadeev et al. 2021). Thus, in this study, high-throughput sequencing of the gut microbiota of AMD was performed using gene sequences derived from this region.

The study revealed that *Firmicutes* and *Bacteroidetes* were the dominant phyla in the gut microbiota of both wild and captive AMD, consistent with other findings in ruminants (Zhang et al. 2018; Qin et al. 2020; Jiang et al. 2022). The relative abundance of the phylum *Bacteroidetes* and genera *Bacteroides*, *Rikenellaceae RC9 gut group*, *NK4A214 group*, and *Alistipes* were significantly higher in captive than wild AMD. The above of them is known to benefit the host by improving their adaptability. *Bacteroides*, a common beneficial bacterial genus, participates in regulating the metabolism of bile acids, short-chain fatty acids (SCFAs), sugars, proteins, and fats. Additionally, *Bacteroides* can also regulate the growth and differentiation of T cells and inhibit the inflammatory response through the secretion of polysaccharide A (Telesford et al. 2015; Zeng et al. 2019), thereby bolstering the host’s immune function. The genus *Alistipes* can also produce SCFAs and butyrate and participates in immune response (Borton et al. 2017). Both *Bacteroides* and *Alistipes* are bile-tolerant microbiota (David et al. 2014), and their relative abundance can be increased by a high-fat diet (Wan et al. 2019). Moreover, the genus *Rikenellaceae RC9 gut group* is related to lipid and amino acid metabolism (Zhou et al. 2018).

In contrast, the abundance of the genera *UCG-005*, *Christensenellaceae R7 group*, *Monoglobus*, *Ruminococcus*, and *Roseburia* in wild AMD was significantly higher than that in captive individuals in this study. These genera are beneficial to host digestion, metabolism, and intestinal homeostasis, which can help wild AMD adapt to harsh conditions in the wild. For example, the genus *Christensenellaceae R7 group* is commonly found in the intestinal tract and mucosa of the host, where it is primarily involved in the degradation of cellulose and metabolism of amino acids, peptides, lipids, and other substances (Waters and Ley 2019), resulting in the production of acetic acid and butyric acid (Tang et al. 2019). The genus *Ruminococcus*, through its secretion of significant amounts of cellulase and hemicellulase, assumes a pivotal role in the degradation and digestion of cellulose and hemicellulose, crucially contributing to food digestion and nutrient metabolism in ruminants (Li et al. 2021a). Furthermore, both of these genera are potentially beneficial bacteria that contribute to the production of SCFAs through the degradation of crude fibers (Wang et al. 2018; Zhang et al. 2020). These SCFAs not only provide energy for the host and gut microbiota but also contribute to the regulation of intestinal mucosal immunity (Peng et al. 2009). The genus *Roseburia* is a marker of gut beneficial bacteria recovery and pathological symptoms (Tamanai-Shacoori et al. 2017) and is involved in plant polysaccharide metabolism and butyrate production (Mack et al. 2016; Di Lodovico et al. 2021). Butyrate, an important energy source for intestinal epithelial cells, promotes intestinal development, maintains the integrity of intestinal epithelial cells, inhibits the growth of pathogenic bacteria and signaling pathways related to inflammatory response, and thus strengthens intestinal microecological defense barriers (Rivière et al. 2016).

Additionally, gene functional annotation and prediction based on KEGG and EggNOG databases showed significant enrichment of metabolic functions related to carbohydrate, amino acid, lipid, and other substances in the gut microbiota of captive AMD compared to wild AMD. These enhanced metabolic functions likely aid in the adaptation of captive AMD to their environment. Captive AMD is primarily fed a diet consisting of roughage and concentrated feed, with the former mainly composed of fruits and leaves, and the latter including corn, soybean, carrot, and other items. Previous studies have demonstrated that metabolic functions increase in captive species (Hale et al. 2019). It is reasonable to assume that the relatively abundant food resources in the captive environment led to a higher relative abundance of *Bacteroides*, *Rikenellaceae RC9 gut group*, and *Alistipes*, as well as other dominant genera of bacteria, which contribute to the metabolic activities of various nutrients. On the other hand, the food resources available to wild AMD are relatively poor. The higher relative abundance of *Firmicutes* and *Christensenellaceae R7 group*, *Ruminococcus*, and *Roseburia* likely contribute to more efficient absorption and utilization of food nutrients in wild individuals. These genera are associated with the production of SCFAs, butyrate, and other small molecules, which can inhibit the growth of pathogenic bacteria, reduce inflammation, and maintain the stability of intestinal microecology in wild AMD.

This study revealed significant differences in gut microbial composition between wild and captive AMD in spring. Forest musk deer (*Moschus berezovskii*) and alpine musk deer (AMD) are closely related species, and previous studies have indicated that there was no significant difference in gut microbial α diversity between wild and captive forest musk deer (Li et al. 2017). In contrast to these findings, the α-diversity of gut microbiota in captive AMD was significantly lower than that in wild AMD, aligning with observations from other studies on ruminants (Gibson et al. 2019). Higher α-diversity of gut microbiota promotes greater complexity and stability, thus enhancing the host’s ability to resist external interference, adapt, and restore equilibrium (Stoffel et al. 2020). Therefore, higher α-diversity is beneficial to the host’s health, while a decrease or loss of α-diversity is closely related to various diseases (Rogers et al. 2016). Long-term feeding environmental conditions and industrialized feed may disrupt the original intestinal microecological balance, leading to a decrease in gut microbial diversity (Sonnenburg and Sonnenburg 2019). Compared with the captive environment, the living environment of wild AMD is relatively harsh but healthier. Therefore, a higher α-diversity of gut microbiota is helpful for wild individuals to adapt to the relatively complex natural environment and resist more threats and adverse factors.

In contrast to published studies on gut microbiota differences between wild and captive AMD (Sun et al. 2020), this study not only examined the variations in potentially pathogenic bacteria but also assessed the stability and complexity of the gut microbiota. Based on the different analyses of potential opportunistic pathogens in AMD, it was found that captive AMD had a higher abundance of the phyla *Actinobacteriota* and *Spirochaetae* and the genera *Odoribacter*, *Streptococcus*, *Oscillibacter*, *Treponema*, *Clostridium *sensu stricto* 1*, *Clostridium *sensu stricto* 6*, *Parasutterella*, *Tyzzerella*, *Bacillus*, *Aerococcus*, and *Corynebacterium*, compared to their wild counterparts. This suggests that captive AMD are more susceptible to intestinal diseases than their wild counterparts. Bacteria of the genus *Odoribacter* are associated with colitis, necrotizing enteritis, and gastroenteritis (Meng et al. 2019). Most bacteria in the genus *Streptococcus* are opportunistic pathogens that can cause intestinal inflammation, produce various neurotoxins, and even lead to bacterial meningitis and permanent neurological damage (Iliev et al. 2009). The anaerobic opportunistic pathogen, *Oscillibacter*, may induce intestinal metabolic dysfunction and metabolic diseases in the host (Hu et al. 2015), while the genus *Parasutterella* is one of the main factors leading to chronic inflammatory states that are related to inflammation (Peng et al. 2019). The genus *Treponema* causes inflammation in the colon and is associated with dysentery, while the genus *Tyzzerella* has been linked to an increased risk of cardiovascular disease (Kelly et al. 2016). The genus *Bacillus* may cause intestinal disorders and acute infections, and the genus *Aerococcus* can lead to meningitis and sepsis, which can pose a potential threat to the host’s health (Li 2004). Most species in the genus *Corynebacterium* are opportunistic pathogens that can cause endocarditis, bacteremia, respiratory tract infections, urinary tract infections, and other types of infections (Aravena-Román et al. 2012). The presence of these potential pathogens may be closely related to the high mortality rate observed in captive AMD.

In contrast, the genera *Erysipelatoclostridium*, *Escherichia-Shigella*, *Anaeroplasma*, *Peptococcus*, *Actinomyces*, and *Ochrobactrum* were found to have higher relative abundance in wild AMD than in captive individuals. The genus *Erysipelatoclostridium* is a conditional pathogen, and its relative abundance is increased in the intestinal tract of patients with gout (Shao et al. 2017). The genus *Escherichia-Shigella* is a typical potential pathogenic bacterium in the intestinal tract. Overgrowth of this genus can cause gut microbiota disorder, which can attack colonic epithelial cells and lead to diarrhea or even intestinal barrier dysfunction (Belotserkovsky and Sansonetti 2018; Zheng et al. 2019). The genus *Peptococcus* is associated with pelvic infections, and its relative abundance is increased in the intestinal tract of patients with inflammatory bowel disease (Zhang et al. 2013). Most species of *Ochrobactrum* are conditional pathogenic bacteria with weak pathogenicity. However, after gene mutation, the genus *Ochrobactrum* can become a highly pathogenic strain with strong infectivity in immunocompromised patients (Teyssier et al. 2007; Arora et al. 2008). The products of this genus may induce host immune dysregulation and interfere with normal neural activities (Li et al. 2021b). It is important to detect and prevent the presence of these pathogenic bacteria and the potential diseases they may cause in captive AMD populations. Gene function annotation analysis based on the KEGG database revealed that the expression levels of disease-related functions were significantly higher in the captive AMD group than in the wild group. This finding suggested that captive individuals may be more susceptible to diseases, and the increased relative abundance of certain potential pathogenic bacteria in the captive groups may further increase the risk of disease.

Although captive breeding is acknowledged as one of the most effective strategies for safeguarding endangered wildlife, prolonged captivity can significantly modify the living environment and dietary composition, resulting in alterations to the composition and functionality of the gut microbiota. Consequently, the reduction in gut microbial diversity can contribute to an elevated proportion of potentially pathogenic bacteria and the enrichment of disease-related functions, posing risks to the well-being of captive individuals. To improve the health status of captive musk deer, we recommend increasing their natural food intake while reducing their reliance on industrialized feed. Additionally, we suggest simulating the natural living conditions of musk deer in the captive environment to help maintain healthy gut microbiota. These results not only provide a theoretical basis for the artificial breeding of musk deer but also offer guidance for assessing the health status of wild populations and the potential success of reintroduction efforts in the future.

## Data Availability

The datasets generated for this study can be found in the NCBI Sequence Read Archive under BioProject PRJNA753621 with the accession number SUB10178189 (https://dataview.ncbi.nlm.nih.gov/object/PRJNA753621?reviewer=cqgndbrcp4dkppi39a3mid6tef).
